# Co-Delivery of Doxorubicin and SATB1 shRNA by Thermosensitive Magnetic Cationic Liposomes for Gastric Cancer Therapy

**DOI:** 10.1371/journal.pone.0092924

**Published:** 2014-03-27

**Authors:** Zhao Peng, Chenxiao Wang, Erhu Fang, Xiaoming Lu, Guobin Wang, Qiang Tong

**Affiliations:** Gastrointestinal Surgery, Union Hospital, Tongji Medical College, Huazhong University of Science and Technology, Wuhan, Hubei, China; Argonne National Laboratory, United States of America

## Abstract

In previous a study, we had developed a novel thermosensitive magnetic delivery system based on liposomes. This study aimed to evaluate the efficiency of this system for the co-delivery of both drugs and genes to the same cell and its anti-tumor effects on gastric cancer. Doxorubicin (DOX) and SATB1 shRNA vector were loaded into the co-delivery system, and in vitro DOX thermosensitive release activity, targeted gene silencing efficiency, targeted cellular uptake, in vitro cytotoxicity, as well as in vivo anti-tumor activity were determined. The results showed that this co-delivery system had desirable targeted delivery efficacy, DOX thermosensitive release and SATB1 gene silencing. Moreover, the co-delivery of DOX and SATB1 shRNA exhibited enhanced activity to inhibit gastric cancer cell growth in vitro and in vivo, compared to single delivery. In conclusion, the novel thermosensitive magnetic drug and gene co-delivery system has promising application in combined chemotherapy and gene therapy for gastric cancer.

## Introduction

Gastric cancer is the fourth common cancer and the second leading cause of cancer death worldwide [1]. In 2008, there were approximately 989,000 new cases of gastric cancer and 738,000 deaths in the world which primarily occurred in East, South, and Central Asia; Central and Eastern Europe; and South America [2], [3]. In China, gastric cancer is the third common cancer with estimated 380,000 new cases and a highest mortality rate about 26.3 per population of 100,000 each year [4], [5]. Surgical resection is the common curative option but it is not suitable for most of patients who are at late stage of gastric cancer. Other therapies such as radiotherapy and chemotherapy show some efficacy, but are often unsatisfactory [4], [6]. In addition, systemic administration of chemotherapy causes adverse effects [7], [8].

Due to the development of drug resistance to chemotherapy, traditional chemotherapy also has exhibited limited anti-tumor efficacy [9]. Therefore, multi-agent co-delivery system has gained more attention recently, because it could deliver different types of agents to the same tumor cells which then exhibit synergistic anti-tumor effects. For example, two different chemical agents can be combined or a chemical agent can be combined with small interfering RNA (siRNA) against an oncogene. Moreover, the targeted delivery and controlled drug release can further enhance anti-tumor effects and reduce adverse effects.

Special AT-rich binding protein (SATB1) is a global chromatin organizer that regulates gene expression and is involved in the modulation of malignant biological behaviors of cancer [10]. Aberrant expression of SATB1 has been shown to promoted breast cancer, lung cancer and lymphoma [11]–[13]. Our previous studies have shown that SATB1 plays an important role in gastric cancer, and may be an independent prognosis marker for gastric cancer [14], [15]. Suppressing the expression of SATB1 by delivering siRNA or plasmid encoding specific interfering short harpin RNA (shRNA) against SATB1 could inhibit the proliferation and invasion, and promote apoptosis of tumor cells [16], [17]. These results suggest that SATB1 is a potential therapeutic target for gastric cancer.

We speculate that the combination of gene therapy and chemotherapy could significantly enhance therapeutic efficacy against gastric cancer. In our previous study, we developed a targeted thermosensitive co-delivery system based on thermosensitive magnetic cationic liposomes (TSMCL). By calcein release assay, we had optimized the thermosensitive liposomal formulation, and then measured the magnetic properties and gene delivery efficiency of TSMCL. In this study, we loaded doxorubicin and SATB1-shRNA vector into this system for the combination of gene therapy and chemotherapy, and evaluated their synergistic anti-tumor effect against gastric cancer in vitro and in vivo.

## Materials and Methods

### Materials

Cholesterol, 1,2-Dipalmitoyl-sn-glycero-3-phosphocholine (DPPC), and 3β-[N-(N',N'-dimethylaminoethane)-carbamoyl]cholesterol (DC-Chol) were purchased from Avanti Polar Lipids (USA). Dimethyldioctadecylammonium bromide (DOAB) were obtained from Sigma-Aldrich (USA). Magnetic Fluid Fe_3_O_4_ was synthesized by Galaxy Nanotech (China). FAM labeled siRNA and plasmid pGFP-SATB1 shRNA were provided by GenePharma (Shanghai, China). Doxorubicin was purchased from Hisun Pharmaceutical (Zhejiang China). Fetal bovine serum (FBS), culture media, penicillin/streptomycin (PEST) and trypsin were supplied from Invitrogen (Carlsbad, CA, USA). SATB1 rabbit monoclonal antibody was from Epitomics (Abcam, UK). All other chemicals were of commercial analytical grade and used without further purification.

### Preparation of co-delivery system

This co-delivery system was based on thermosensitive cationic liposomes, which were prepared with a thermosensitive cationic formulation of DPPC, DC-Cholesterol, DOAB and Cholesterol at a molar ratio of 80∶5∶5∶10 optimized in our preliminary experiments. Liposomes(TSCL) were prepared by the thin film hydration method, followed by extrusion [18]. The ammonium sulfate gradient method was used to load DOX into TSCL (TSCL-DOX). To prepare magnetic liposomes (TSMCL), magnetic fluid Fe_3_O_4_ was used as the core and co-encapsulated with ammonium sulfate buffer into the liposomes. After the formation of thin film in the round bottom flask, one milliliter of suspension of iron and ammonium sulfate was added to hydrate the film, and then extruded through the polycarbonate filters and loaded with DOX (TSMCL-DOX) by the ammonium sulfate gradient method. Finally, the products from the gel filtration were centrifuged at 1,000 g for 15 min to remove unencapsulated Fe_3_O_4_.

pGFP-SATB1-shRNA (shSATB1) vector was incubated with different liposomes in serum free media at room temperature for 30 min, to prepare TSCL-shSATB1, TSCL-DOX-shSATB1, TSMCL-shSATB1 and TSMCL-DOX-shSATB1, respectively.

### Determination of zeta potential, particle size, and polydispersity

The average particle size and the polydispersity of the particle-size distribution of the liposomes were determined at 25°C by dynamic light scattering using a ZetaPALS particle sizing instrument (Brookhaven Instruments Corporation, USA). The zeta potential of the liposomal dispersions was measured using the same instrument at 25°C by the electrophoretic mobility.

### Determination of Doxorubicin loaded efficiency

DOX concentration in the liposomes was measured by a fluorimeter (Perkin Elmer, USA) with 485 nm excitation and 590 nm emission filters with a dilution series of free DOX as the standard. DOX loaded efficiency was calculated based on DOX concentration.

### Differential Scanning Calorimetry (DSC)

DSC was performed to evaluate the thermosensitivity of liposomes by determining the phase transition temperature (Tm). Tm was evaluated using a nano DSC (TA Instruments, USA) at a heating rate of 20°C/h with 20 mg/ml phospholipid.

### In vitro thermosensitive DOX release assay

20 μl DOX loaded liposomes were incubated in 1 ml PBS and PBS with 50% fetal bovine serum (FBS) which mimics the in vivo environment separately in Eppendorf tubes. The samples were heated in water bath at 37°C and 42°C for 1 h, respectively. Then the fluorescence intensity of DOX was measured in a fluorimeter (Perkin Elmer, USA) using 485 nm excitation and 590 nm emission filters. For 100% release, samples were incubated in 1 ml PBS with 1% Triton X-100 for 1 min. The DOX release percentages were calculated as followed:
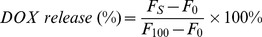
where F_s_ was the fluorescence of samples after heating, F_0_ was the initial fluorescence of samples before heating, and F_100_ was the fluorescence of samples treated with Triton X-100.

### Cell culture

Human gastric adenocarcinoma MKN-28 cell line was purchased from KeyGEN Biotech (Nanjing, China) and cultured in high-glucose DMEM supplemented with 10% FBS, 100 U/mL penicillin and 100 μg/mL streptomycin in a humidified atmosphere of 5% CO_2_ at 37°C.

### In vitro cell transfection

MKN-28 cells were seeded in 12-well plates at a density of 4×10^5^ cells/well and grown overnight to approximately 80% confluence. Next day, the cells were washed twice with pre-warmed PBS, then TSCL-shSATB1 and TSMCL-shSATB1 were added into each well and incubated for 6 h. Cells incubated with TSMCL-shSATB1 were positioned on 12-well magnetic plate during the first 30 min to offer a magnetic field. Next, the incubation medium was replaced with DMEM supplemented with 10% FBS and the cells were incubated for 24 h. GFP expression level was evaluated under fluorescence microscope (Nikon 80i) and flow cytometer (BD FACS Canto II).

### Real-time quantitative polymerase chain reaction (RT-qPCR)

Total RNA was extracted from MKN-28 cells using TRIzol regent (Invitrogen, Carlsbad, CA) following the manufacturer's instruction, and cDNA was synthesized using PrimeScript RT Master MIX (Takara, Dalian). PCR was performed using SYBR Premix Ex Taq II (Takara, Dalian) on a StepOne Plus Real-Time PCR System (Applied Biosystems, USA). The sequences of the primers were as follows: SATB1 sense 5′-ACAGAACCCTGTGGGAGAAC-3′, and antisense 5′-GCGTTGCTCTCCTGTTCATA-3′ GADPH sense 5′-ACAGAACCCTGTGGGAGAAC-3′, and antisense 5′-GCGTTGCTCTCCTGTTCATA-3′. SATB1 mRNA level was normalized to that of GAPDH.

### Western blot analysis

MKN-28 cells were harvested and lysed in RIPA buffer. The supernatants were collected after centrifugation at 10,000 g for 10 min. Protein concentration of the supernatant was determined using a BCA Protein Assay Kit. Then 40 μg of samples were run on a 15% SDS-PAGE and proteins were transferred to PVDF membranes. Next, the membranes were incubated in 5% non-fat milk for 1 h to block non-specific binding and then incubated overnight at 4°C with SATB1 or β-actin antibody (1∶500 dilution). The membranes were then probed with HRP-conjugated goat anti rabbit secondary antibody for 30 min. Finally, the membranes were visualized with an enhanced chemiluminescence system (ECL, Pierce) and exposed to X-ray film.

### In vitro evaluation of cellular uptake

To evaluate the intracellular location of delivered DOX and pGFP-SATB1 shRNA and the enhanced penetration of liposomes into the gastric cancer cells by magnetic targeted, a FAM labeled siRNA (green fluorescence) was used to imitate pGFP-SATB1 shRNA, and DOX by itself possesses an instinct red fluorescence to monitor cellular uptake. MKN-28 cells were seeded in 12-well plate at 4×10^5^ cells/well and grown overnight. Then the cells were incubated with free siRNA, TSCL-siRNA, TSMCL-siRNA, TSCL-DOX, TSMCL-DOX, TSCL-DOX-siRNA or TSMCL-DOX-siRNA for 6 h. For magnetic liposomes, the plates were positioned on a 12-well magnetic plate during the first hour of incubation. The cells were visualized under fluorescence microscope to locate the fluorescent labels of DOX and siRNA. The nuclei were stained with DAPI (blue fluorescence).

### MTT assay

MKN-28 cells were seeded at a density of 2×10^4^ cells/well in 96-well plates and grown overnight. The cells were then incubated with different liposomes at 37°C for 2 h in CO_2_ incubator. For magnetic liposomes, the plates were placed under a 96-well magnetic plate during the first 1 h of incubation. Next the cells were washed twice with PBS and incubated for 46 h in fresh media. Subsequently, the cell viability was measured by MTT assay. The medium in each well was replaced by 20 μl MTT solution (Sigma-Aldrich, USA) and incubated for 4 h at 37°C, then the supernatants were discarded and formazan crystals were dissolved with 200 μl DMSO. The plates was measured at 490 nm in a microplate reader, and the cell viability was calculated according to the following formula:
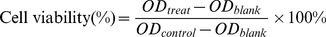



### Flow cytometry

Apoptosis was detected using an Annexin V-FITC Apoptosis Detection Kit (eBioscience, USA). MKN-28 cells were seeded in 12-well plates and treated as described above. After 24 h, the cells were collected, washed twice with PBS and suspended in 500 μl binding buffer. Next the cells were double stained with Annexin V-FITC and propidium iodide (PI). Cells in early stage of apoptosis stained with Annexin V-FITC but not stained with PI were quantified by flow cytometery.

### Xenograft mouse model

Five to six weeks old male Balb/c nude mice were provided by the Center for Animal Experiments of Tongji Medical College. Each mouse was inoculated subcutaneously into the right flank with 5×10^6^ MKN-28 cells. Several weeks after tumor inoculation, 36 tumor bearing mice were randomly divided into six groups (n = 6). The mice were injected via tail vein with Free DOX, TSMCL-DOX, TSMCL-shSATB1, TSCL-DOX-shSATB1, TSMCL-DOX-shSATB1 or normal saline as control. The dose of DOX was 2.5 mg/kg and that of pGFP-SATB1 shRNA was 10 μg per mouse. The treatment was given once every 3 days and tumor size was measured via a calipering and then tumor volume could be calculated by the following formula: volume = (D_Min_)^2^×D_Max_/2, where D_Max_ was the longest tumor diameter and D_Min_ was the shortest one. For TSMCL-DOX, TSMCL-shSATB1 and TSMCL-DOX-shSATB1, the magnetic targeting was achieved using a continued external magnetic field of 5000 Gauss for 30 min focusing on the tumor after drug administration. All experiments were approved by Ethics Committee of Tongji Medical College and all animals were treated humanely according to Institutional Animal Care and Use Committee guidelines.

### Statistic analysis

Data were expressed as the mean ± standard deviation (SD). The statistical significance between different groups was evaluated with Student's t-test and one-factor analysis of variance (ANOVA) test. For the survival time of the animals, the Kaplan-Meier curves of each group were established and log rank test was performed to compare survival rate. p<0.05 was considered statistically significant.

## Results

### Characterization of liposomes

As shown in Table 1, the diameter of TSCL was 83.6±5.7 nm, while that of TSCL-DOX was increased to 118.5±7.9 nm due to the encapsulation of DOX. Meanwhile, the diameters of TSCL-shSATB1 and TSCL-DOX-shSATB1 were increased significantly to 161.1±11.8 nm and 238.1±20.6 nm, respectively, due to the adhesion and fusion of plasmid DNA to liposomes. For magnetic liposomes, the particle sizes of TSMCL, TSMCL-DOX, TSMCL-shSATB1 and TSMCL-DOX-shSATB1 were 135.0±11.6 nm, 157.2±14.3 nm, 221.3±15.7 nm and 319.4±20.1 nm, respectively, significantly larger than that of TSCL, TSCL-DOX, TSCL-shSATB1 and TSCL-DOX-shSATB1 due to the entrapment of the magnetic nanoparticles. Furthermore, the size of TSMCL-shSATB1 and TSMCL-DOX-shSATB1 was significantly larger that of TSMCL and TSMCL-DOX, respectively (p<0.05). All liposomes had narrow size distribution because their polydispersities were no more than 0.3.

**Table 1:** Particle size, polydispersity and zeta potential of liposomes.

Group	Particle size (nm)	Polydispersity	Zeta potential (mV)
TSCL	83.6±5.7	0.212±0.023	+52.0±7.3
TSCL-DOX	118.5±7.9	0.255±0.017	+50.1±7.7
TSCL-shSATB1	161.1±11.8#	0.174±0.014	+31.3±5.2#
TSCL-DOX-shSATB1	238.1±20.6#	0.241±0.031	+26.7±4.5#
TSMCL	135.0±11.6*	0.304±0.002	+45.3±5.7
TSMCL-DOX	157.2±14.3*	0.225±0.037	+42.7±4.4
TSMCL-shSATB1	221.3±15.7*,#	0.289±0.033	+26.1±6.5#
TSMCL-DOX-shSATB1	319.4±20.1*,#	0.314±0.027	+19.8±3.1#

The data were expressed as mean±SD (n = 3).

# p <0.05 TSCL-shSATB1 vs. TSCL,TSCL-DOX-shSATB1 vs. TSCL-DOX, TSMCL-shSATB1 vs. TSMCL,TSMCL-DOX-shSATB1 vs. TSMCL-DOX,

*p <0.05 TSMCL vs. TSCL, TSMCL-DOX vs. TSCL-DOX, TSMCL-shSATB1 vs. TSCL-shSATB1 and TSMCL-DOX-shSATB1 vs. TSCL-DOX-shSATB1.

Zeta potentials of TSCL, TSCL-DOX, TSCL-shSATB1 and TSCL-DOX-shSATB1 were 52.0±7.3 mV, 50.1±7.7 mV, 31.3±5.2 mV and 26.7±4.5 mV, respectively. After incubation with pGFP-SATB1- shRNA, the surface charges decreased significantly (p<0.05), owing to the electrostatic interaction between the cationic lipids and plasmid DNA. However, entrapment of magnetic nanoparticles did not result in a significant decrease of Zeta potential in magnetic liposomes.

For DOX loaded efficiency, the DOX encapsulating rate was 89±3% and 78±8% (n = 3) for TSCL-DOX and TSMCL-DOX, respectively, and was 85±7% and 73±9% (n = 3) for TSCL-DOX-shSATB1 and TSMCL-DOX-shSATB1, respectively. These data indicate that the interaction between liposomes and plasmid did not result in DOX leakage.

### The thermosensitivity of the liposomes

**Figure 1:**
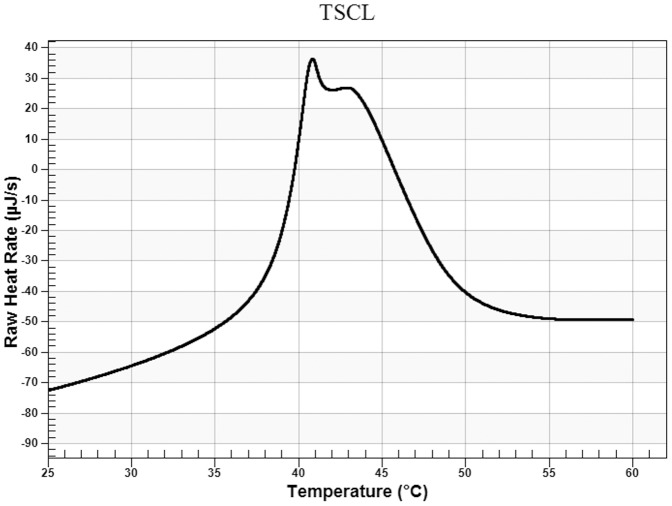
Differential scanning calorimetric scan (DSC) analysis of TSCL (DPPC∶DOAB∶DC-Cholesterol∶Cholesterol 80∶5∶5∶10) (molar ratio).

Differential Scanning Calorimetry was performed to determine the phase transition temperature of TSCL. As shown in Fig. 1, TSCL composed of DPPC, DC-Cholesterol, DOAB and Cholesterol at a molar ratio of 80∶5∶5∶10 had a Tm of 40.8°C with a relatively broader transition peak which may be the fusion transition peak of DPPC and DOAB.

### In vitro thermosensitive DOX release from the liposomes

**Figure 2:**
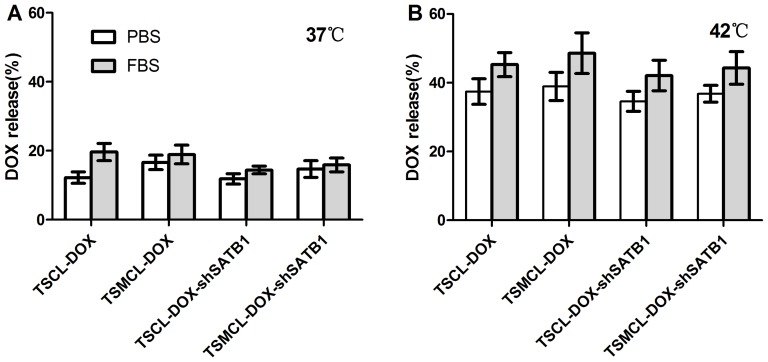
DOX release from different liposomes. The release of DOX from different liposomes at 37°C (**A**) and 42°C (**B**) after incubation with PBS or 50% FBS. The values were expressed as mean±SD (n = 3).

As shown in Fig. 2, DOX release rate from TSCL-DOX and TSMCL-DOX was only 12% and 16% after incubation with PBS at 37°C, and increased to 20% and 19% after incubation with 50% FBS, respectively. For TSCL-DOX-shSATB1 and TSMCL-DOX-shSATB1, DOX release rate was 12% and 15% after incubation with PBS at 37°C, and was both 16% after incubation with 50% FBS, indicating that the incorporation of plasmid had no significant effect on the stability of liposomes (p>0.05).

DOX release rate from TSCL-DOX and TSMCL-DOX was increased to 37% after incubation with PBS at 42°C, and increased to 45% and 49% after incubation with 50% FBS, respectively. For TSCL-DOX-shSATB1 and TSMCL-DOX-shSATB1, DOX release rate was 35% and 43% after incubation with PBS and FBS at 42°C, respectively, both significantly higher than that at 37°C (p<0.05). These results indicate that TSCL-DOX, TSMCL-DOX, TSCL-DOX-shSATB1 and TSMCL-DOX-shSATB1 have desirable thermosensitivity, and could be used for hyperthermia triggered control release of DOX.

### Silencing of SATB1 expression in MKN-28 cells transfected with liposomes

**Figure 3:**
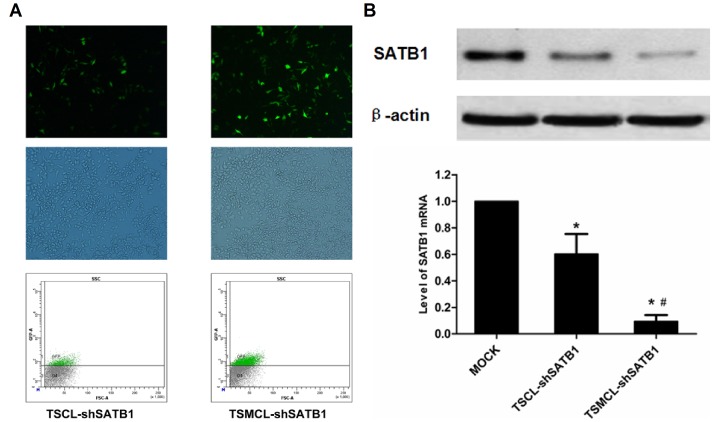
Silencing of SATB1 expression by different liposomes. (**A**) Fluorescent imaging of MKN-28 cells transfected by TSCL-shSATB1 and TSMCL-shSATB1. (**B**) Quantitative analysis of transfection efficiency of TSCL-shSATB1 and TSMCL-shSATB1 by FACS. (**C**) Western blot analysis of SATB1 protein level in MKN-28 cells transfected by TSCL-shSATB1 and TSMCL-shSATB1. (**D**) Real-time PCR analysis of SATB1 mRNA level in MKN-28 cells transfected by TSCL-shSATB1 and TSMCL-shSATB1. The values were expressed as mean±SD from three independent experiments. * p<0.05 compared with mock control; # p<0.05 compared with TSCL-shSATB1.

Next we evaluated the efficiency of the liposomes to deliver shSATB1 vector into MNK-28 cells. Typical fluorescence images of cells transfected with TSCL-shSATB1 and TSMCL-shSATB1 were shown in Fig. 3A. Flow cytometry analysis showed that the transfection efficiency of TSCL-shSATB1 was only 15.4±0.15%. However, after the application of magnetic field guidance, the transfection efficiency of TSMCL-shSATB1 was 34.3±0.93%, significantly higher than that of TSCL-shSATB1 (Fig. 3B).

To determine whether the delivered shSATB1 vector could mediate silencing of SATB1 expression in MKN-28 cells, we performed Real-time quantitative PCR and Western blot analysis. The results showed that both SATB1 mRNA and protein levels decreased in cells transfected with TSCL-shSATB1 and TSMCL-shSATB1, compared to control cells. Moreover, TSMCL-shSATB1 with the help of magnetic field was more potent than TSCL-shSATB1 to inhibit SATB1 expression in MKN-28 cells (Fig. 3C, D).

### Magnetic targeted in vitro cellular uptake

**Figure 4:**
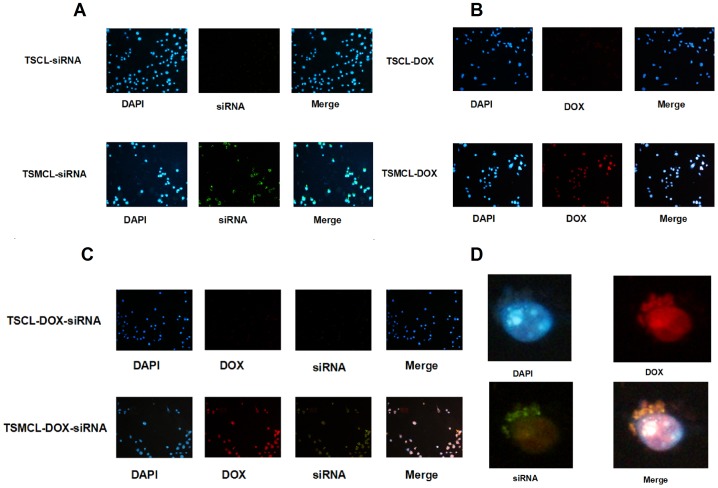
Uptake of DOX and FAM-labeled siRNA by MKN-28 cells. (**A**) Imaging of cells after treatment with TSCL-siRNA and TSMCL-siRNA. (**B**) Imaging of cells after treatment with TSCL-DOX and TSMCL-DOX. (**C**) Imaging of cells after treatment with TSCL-DOX-siRNA and TSMCL-DOX-siRNA. (**D**) Typical detailed imaging of the location of DOX and siRNA in cells after treatment with TSMCL-DOX-siRNA.

To compare the penetrations into the cells between non-magnetic and magnetic liposomes with the application of magnetic targeted guidance, as well as the intracellular location of delivered DOX and shSATB1, a FAM-labeled siRNA was used as the indicator. The absence of green fluorescence in cells treated with Free siRNA indicated that it could not penetrate into the cells (data not shown). We observed that more cells treated with TSMCL-siRNA exhibited green fluorescence than cells treated with TSCL-siRNA (Fig. 4A). Moreover, more cells treated with TSMCL-DOX showed red fluorescence than cells treated with TSCL-DOX (Fig. 4B). In cells treated with TSMCL-DOX-siRNA, high fluorescence intensity was observed, and the cells appeared pink due to the merging of blue, red and green color (Fig. 4C). These observations suggest that TSMCL is more potent in delivering siRNA and DOX into the cells than TSCL, after the application of magnetic field.

Furthermore, we examined the intracellular location of delivered DOX and siRNA. Red fluorescence was observed in both the nuclei and the cytoplasm, indicating that delivered DOX was located in both the nuclei and the cytoplasm. In contrast, green fluorescence primarily appeared in the cytoplasm, suggesting that the siRNAs were delivered into the cytoplasm (Fig. 4D). The merging of red and green appeared yellow in the cytoplasm, and the merging of blue and red appeared lavender in the nuclei. Taken together, these data indicate that both TSCL and TSMCL can penetrate into gastric cancer cells and deliver their contents into the cytoplasm (DOX and siRNA) and the nuclei (DOX).

### In vitro anti-tumor effects of the liposomes

**Figure 5:**
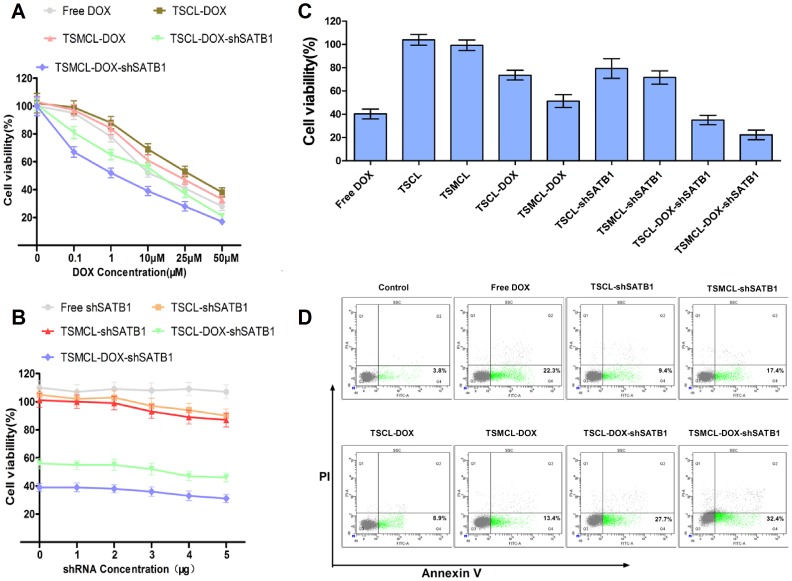
In vitro anti-tumor effects of different liposomes to MKN-28 cells. (**A**) The viability of cells after treatment with liposomes loaded with different concentrations of DOX. (**B**) The viability of cells after treatment with liposomes loaded with different concentrations of shSATB1. (**C**) The viability of cells after treatment with liposomes as indicated. The values were expressed as mean±SD (n = 3). (**D**) The apoptosis of cells after treatment with liposomes as indicated.

We evaluated the in vitro anti-tumor effects of this co-delivery system by cytotoxicity and apoptosis induction activity. The cytotoxicity of the liposomes was assessed by MTT assay. To determine the effects of DOX and plasmid concentrations on the cytotoxicity of liposomes, we examined liposomes loaded with various concentrations of DOX and different amounts of SATB1 shRNA. With the increase of DOX concentration, the cytotoxicity of the liposomes was increased (Fig. 5A). However, the addition of SATB1 shRNA concentration did not result in increased cytotoxicity, and there were only slightly increase of cytotoxicity when SATB1 shRNA concentration was about 4 μg (Fig. 5B). Thus we used 25 μM DOX and 4 μg SATB1 shRNA to compare the cytotoxicity among Free DOX, Free shRNA, TSCL, TSMCL, TSCL-DOX, TSMCL-DOX, TSCL-shSATB1, TSMCL-shSATB1, TSCL-DOX-shSATB1 and TSMCL-DOX-shSATB1.

As shown in Fig. 5C, free shRNA, TSCL and TSMCL had little cytotoxicity. Cell viability was 40.3±3.4%, 73.6±3.5% and 51.3±4.5% for free DOX, TSCL-DOX and TSMCL-DOX, respectively, suggesting that cytotoxicity of TSMCL-DOX was higher than TSCL-DOX, but lower than free DOX. Cell viability was 79.2±6.9% and 71.6±4.7% for TSCL-shSATB1 and TSMCL-shSATB1, respectively, showing no statistic significance (p>0.05). For drug and gene co-delivery, cell viability was only 35.0±3.2% and 22.3±3.4% for TSCL-DOX-shRNA and TSMCL-DOX-shRNA, respectively, significantly lower than that of Free DOX, TSCL-DOX, TSMCL-DOX and SATB1shRNA loaded liposomes (p<0.05). In addition, cell viability of TSMCL-DOX-shRNA was significantly lower than that of TSCL-DOX-shRNA (p<0.05). These results suggest that a synergistic cytotoxicity effect is achieved by co-delivering DOX and SATB1 shRNA. Moreover, with the application of magnetic targeted, a further enhanced cytotoxicity can be obtained.

To determine the additional anti-tumor mechanism besides the cytotoxicity of the co-delivery system, we examined the apoptosis rate of MKN-28 cells treated with Free DOX, TSCL-DOX, TSMCL-DOX, TSCL-shSATB1, TSMCL-shSATB1, TSCL-DOX-shSATB1 and TSMCL-DOX-shSATB1 by flow cytometry. As showed in Fig. 5D, apoptosis rate was 22.3% in cells treated with Free DOX, was 8.9% in cells treated with TSCL-DOX, but increased to 13.4% in cells treated with TSMCL-DOX and magnetic field. Similarly, apoptosis rate was 9.4% in cells treated with TSCL-shSATB1, but increased to 17.4% in cells treated with TSMCL-shSATB1 and magnetic field. In contrast, apoptosis rate was 27.7% in cells treated with TSCL-DOX-shSATB1, higher than that in cells treated with TSCL loaded with DOX or shSATB1 alone. The apoptosis rate was 32.4% in cells treated with TSMCL-DOX-shSATB1, the highest among all groups. These results demonstrate that co-delivering DOX and SATB1 shRNA leads to combined effects of apoptosis induction. In a word, this co-delivery system exhibits strong anti-tumor effects in vitro.

### In vivo anti-tumor activity of the liposomes

**Figure 6:**
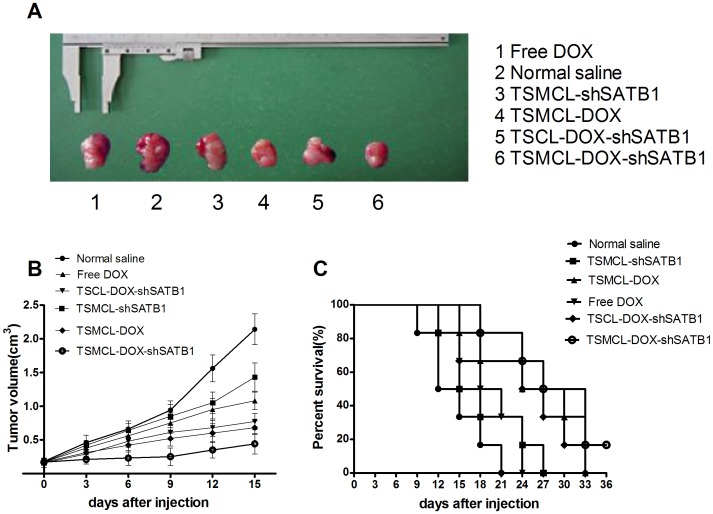
In vivo anti-tumor activity of different liposomes. (**A**) Typical imaging of xenografted tumor. (**B**) Tumor size after treatment with Free DOX, TSMCL-DOX, TSMCL-shSATB1, TSCL-DOX-shSATB1, or TSMCL- DOX-shSATB1, treatment with normal saline was used as control. Data were presented as mean±SD (n = 6). (**C**) Survival curves of tumor bearing mice treated with Free DOX, TSMCL-DOX, TSMCL-shSATB1, TSCL-DOX-shSATB1, or TSMCL- DOX-shSATB1, treatment with normal saline was used as control.

In order to determine the in vivo anti-tumor activity of the co-delivery system, we established MKN-28 murine xenograft models and injected Free DOX, TSMCL-DOX, TSCML-shSATB1, TSCL-DOX-shSATB1 or TSMCL-DOX-shSATB1 into the mice through tail vein. The treatment was given once every 3 days, and the tumors were dissected (Fig. 6A). On day 15, tumor volume was 0.44±0.05 cm^3^ in mice treated with TSMCL-DOX-shSATB1, significantly lower than that in saline group (2.14±0.23 cm^3^), Free DOX group (1.08±0.13 cm^3^), TSMCL-DOX group (0.68±0.10 cm^3^), TSCML-shSATB1 group (1.43±0.21 cm^3^), and TSCL-DOX-shSATB1 group (0.77±0.12 cm^3^) (Fig. 6B).

Moreover, as shown in Fig. 6(C), the median time for tumor volume to reach 2 cm^3^ was 30 days in TSMCL-DOX-shSATB1 group, longer than that in saline group, Free DOX group, TSMCL-DOX group, TSCML-shSATB1 group and TSCL-DOX-shSATB1 group (Fig. 6C). Taken together, these results indicate that co-delivering DOX and shSATB1 by TSMCL exhibits synergistic anti-tumor effect in vivo.

## Discussion

Despite recent development in surgery, radiotherapy and target therapy, chemotherapy is still one of the important approaches for gastric cancer therapy. However, therapeutic effects of chemotherapy are often unsatisfied and could not significantly improve the prognosis of cancer patients [19]. One main reason is the tumorigenesis and progression of gastric cancer involves a variety of different mechanisms; the unitary anti tumor mechanism of traditional chemotherapy has limited their therapeutic effects, thus the combination of chemotherapy with gene therapy may improve anti-tumor effects. Another obstacle of chemotherapy is that systemic drug administration leads to limited drug concentration in tumor sites while causing many adverse effects [8]. The key to solve these problems relies on new manners of drug delivery, therefore, developing targeted multi-agents delivering system that can be directly guided to the tumor site with controlled release can overcome these problems and enhance therapeutic effects [20]–[25].

Among various drug delivery systems, liposomes are most promising for good biocompatibilities that cause little or no antigenic, allergic, and toxic reactions, and easily undergo biodegradation. As both drug and gene carriers, liposomes can not only protect the host from undesirable effects of the encapsulated drug, but also prevent the entrapped contents from premature inactivation by the physiological medium [26]. Moreover, liposome is a potentially targeted drug delivery system, whether it is achieved by enhanced permeability and retention (EPR) effect (Passive targeted), or by magnetic field guidance and immune connection (Active targeted) [27]. In addition, some liposomes possess controlled triggered drug release features, such as thermo sensitivity, pH sensitivity and microwave sensitivity.

Recently we had developed a novel magnetic targeted thermosensitive drug and gene co-delivery system (TSMCL). Based on an electroneutral thermosensitive formulation (DPPC∶Cholesterol = 80∶20), we had added different cationic components and optimized the thermosensitivity of liposomes by calcein release assay. The results had indicated that we could get a desirable thermosensitivity by replacing 10 mol parts Cholesterol of 5 mol parts DC-Cholesterol and 5 mol parts DOAB (DPPC∶DC-Cholesterol∶DOAB∶Cholesterol = 80∶5∶5∶10), and calcein release from the liposomes would be lower at 37°C but significant higher at 42°C in this formulation. Next, Magnetic fluid Fe_3_O_4_ had been used as the core and functioned as magnetic targeting and heating source of TSMCL. Vibrating Sample Magnetometer (VSM) measurement had indicated that magnetic fluid Fe_3_O_4_ had been superparamagnetic, thus TSMCL would have good magnetic targeted effects. Meanwhile, the time-dependent heating curve also had shown that both magnetic fluid Fe_3_O_4_ and TSMCL could be heated from 25°C to 42°C within 20 min. With the help of Magnetic fluid Fe_3_O_4_, both magnetic targeting and temperature triggered drug release of TSMCL could be realized. Finally, both TSCL and TSMCL had exhibited typical liposomal morphologies and good distributions under TEM. Based on the successful construction of TSMCL, in this study we loaded DOX and SATB1 shRNA vector into TSMCL to make TSMCL-DOX-shSATB1 and evaluated the anti-tumor effects against gastric cancer cells in vitro and in vivo.

DOX is a commonly used drug in chemotherapy with high efficiency to inhibit tumor cell proliferation and induce tumor cell apoptosis, but its therapeutic effects are limited due to severe cardiotoxicity and myelosuppression when administrated systemically [28]. Although DOX liposomes have partially improved the situation, the lack of targeted delivery still limites their utilization [29]. SATB1 is a global chromatin organizer that directly regulates the expression of ERRB2, MMP2, ABL1 and E-cadherin to act as a key regulator of cancer development [30]. Overexpression of SATB1 in various tumors has been associated with malignant biological behaviors such as invasion, proliferation and metastasis [10]–[13]. Silencing SATB1 expression by small interfering RNA (siRNA) or plasmid encoding short harpin RNA (shRNA) could inhibit, the proliferation, invasion and metastasis, and induce apoptosis of various tumor cells [16], [17]. Therefore, SATB1 becomes a potential target for cancer therapy [30]. However, appropriate delivery vectors for siRNA or shRNA are important for SATB1 targeted cancer gene therapy.

In this study, using TSMCL-DOX-shSATB1 system, both DOX and SATB1 shRNA vector could be guided to tumor site under magnetic filed guidance, and DOX was released in a hyperthermia triggered manner. Hyperthermia triggered release is dependent on thermosensitive liposomes which are primarily composed of Dipalmitoyphosphocholine (DPPC) which undergoes a gel to liquid crystalline phase transition (Tm) that becomes highly leaky to small water-soluble molecules at 41°C [31]. Liposomes composed of different DPPC and lipids have distinct Tm and thermosensitivity [32]. DSC analysis showed that the Tm of our delivery system was 40.8°C, and DOX release assay indicated that it was steady at 37°C while DOX was released when the temperature raised to 42°C. Moreover, the loading of shSATB1 had no significant effects on thermosensitive release of DOX. Therefore, our liposomal formulation exhibits desirable thermosensitivity and can be used for hyperthermia trigger control release in combination with local thermal therapy. However, thermosensitive drug release results from the increase in the permeability of liposomes, rather than liposomes burst, and the incorporation of cholesterol (to increase serum stability) may have negative effects on the thermosensitivity of liposomes by broadening the transition peak [33], [34]. Therefore, even though we obtained desirable thermosensitivity, the drug release was still relatively low.

Magnetic drug targeting (MDT) has been developed as drug carrier to promote drug accumulation in the targeted tumor site under magnetic field guidance [35], [36]. Meanwhile, gene delivery efficiency could be improved with magnetic cationic liposomes known as liposomal magnetofection [37]–[39]. In this study we designed TSMCL-DOX-shSATB1 as a MDT and magnetofection combined delivery system to enhance DOX and SATB1 shRNA delivery efficiency. Cellular uptake experiment indicated that enhanced delivery efficiency was achieved with magnetic field guidance, whether for DOX, for shSATB1, or for both, Analysis of SATB1 expression showed that TSMCL-shSATB1 had better efficiency of silencing SATB1 expression than TSCL-shSATB1. Furthermore, MTT assay showed that TSMCL-DOX, TSMCL-shSATB1 and TSMCL-DOX-shSATB1 had higher cytotoxicity to gastric cancer cells compared with TSCL-DOX, TSCL-shSATB1 and TSCL-DOX-shSATB1, respectively, and this is correlated to higher apoptosis rates in the cells with magnetic targeting. Lastly, in vivo murine xenograft models showed that co-delivery of DOX and shSATB1 vector exhibited stronger effects in inhibiting tumor growth with magnetic targeting. Collectively, these data confirm that enhanced delivery efficiency and anti-tumor activity can be achieved with magnetic targeting.

In summary, our study showed that the novel drug and gene co-delivery system exhibits combined features of magnetic targeting, thermosensitive control release and synergistic anti-tumor effects in vitro and in vivo. Therefore, this thermosensitive magnetic targeted co-delivery system has potential application in combined chemotherapy and gene therapy for gastric cancer.
